# Interventions Made to Preserve Cognitive Function Trial (IMPCT) study protocol: a multi-dialysis center 2x2 factorial randomized controlled trial of intradialytic cognitive and exercise training to preserve cognitive function

**DOI:** 10.1186/s12882-020-02041-y

**Published:** 2020-09-03

**Authors:** Mara A. McAdams-DeMarco, Nadia M. Chu, Malu Steckel, Sneha Kunwar, Marlís González Fernández, Michelle C. Carlson, Derek M. Fine, Lawrence J. Appel, Marie Diener-West, Dorry L. Segev

**Affiliations:** 1 Department of Surgery, Johns Hopkins University School of Medicine, Baltimore, MD USA; 2 Department of Epidemiology, Johns Hopkins Bloomberg School of Public Health, 615, N. Wolfe St, W6033, Baltimore, MD 21205 USA; 3 Department of Physical Medicine and Rehabilitation, Johns Hopkins University School of Medicine, Baltimore, MD USA; 4 Department of Mental Health, Johns Hopkins University School of Medicine, Baltimore, MD USA; 5 Department of Medicine, Johns Hopkins University School of Medicine, Baltimore, MD USA; 6 Department of Biostatistics, Johns Hopkins Bloomberg School of Public Health, Baltimore, MD USA

**Keywords:** intradialytic exercise, hemodialysis, cognitive function, executive function

## Abstract

**Background:**

Kidney disease and dialysis significantly impact cognitive function across the age spectrum. Cognitive training (CT) and/or exercise training (ET) are promising approaches to preserve cognitive function among community-dwelling older adults, but have not been tested for cognition preservation in hemodialysis patients of all ages. In this manuscript, we summarize the protocol for the Interventions Made to Preserve Cognitive Function Trial (IMPCT).

**Methods:**

We will perform a 2 × 2 factorial randomized controlled trial (RCT) of eligible adult (≥18 years) hemodialysis initiates (*n* = 200) to test whether intradialytic CT (brain games on a tablet PC), ET (foot peddlers) and combined CT + ET while undergoing hemodialysis preserves executive function compared to standard of care (SC). Participants will engage in the interventions to which they are randomized for 6 months. The primary objective is to compare, among interventions, the 3-month change in executive function measured using the Trail Making Test A (TMTA) and B (TMTB); specifically, executive function is calculated as TMTB-TMTA to account for psychomotor speed. This primary outcome was selected based on findings from our pilot study. The secondary objectives are to compare the risk of secondary cognitive outcomes, ESKD-specific clinical outcomes, and patient-centered outcomes at 3-months and 6-months. All data collection and interventions are conducted in the dialysis center.

**Discussion:**

We hypothesize that receiving intradialytic CT or ET will better preserve executive function than SC but receiving combined CT + ET, will be the most effective intervention. The current trial will be an important step in understanding how intradialytic interventions might preserve cognitive health.

**Trial Registration:**

Clinicaltrials.Gov (Date: 8/6/18): # NCT03616535. Protocol Version: 10 (April 2020). Funding: NIDDK R01DK114074.

## Background

Over 640,000 adults in the US suffer from end-stage kidney disease (ESKD) [1] and over 95% of newly diagnosed patients initiate hemodialysis. Hemodialysis is often the only long-term treatment option for ESKD patients [1]. Hemodialysis is performed at a minimum of 3 times a week for at least 4–6 h per session and continues for the patient’s lifetime or until successful kidney transplantation. However, given the kidney transplantation waiting list of nearly 100,000 patients, a substantial amount of patient time can be spent on hemodialysis while waiting for KT [2]. In a study of 431 patients undergoing hemodialysis, 87.9% reported watching television and 72.4% reported sleeping during the dialysis session; participating in these passive intradialytic activities was associated with worse health-related quality of life (HRQOL), particularly mental and kidney disease-specific HRQOL [3]. Therefore, it is likely that the time spent on hemodialysis may be a missed opportunity to improve the health of patients with ESKD.

Cognitive decline and dementia [4–6] are well-recognized complications of ESKD and hemodialysis [7, 8]. One study suggested that only 13% of patients undergoing hemodialysis have normal cognitive function [9]. Many patients already have partially compromised cognitive function upon initiating hemodialysis [10–12], and cognitive function declines at an accelerated rate [13] while undergoing hemodialysis [14]. In fact, patients undergoing hemodialysis suffer from a 3-fold higher rate of cognitive impairment than age-matched controls [15]. In the hemodialysis population, poor cognitive function is not limited to older adults but occurs across the age spectrum [16–20].

Among patients undergoing hemodialysis, cognitive impairment is associated with poor medication compliance, increased number of hospitalizations [7], increased mortality [15, 21], and decreased access to kidney transplantation [17, 19]. Severe cognitive impairment is problematic as it impedes patients’ ability to comply with their dialysis schedule, maintain complicated medication regimens for chronic conditions, retain the capacity for self-care, make informed decisions, and adhere to fluid and dietary restrictions [7].

In community-dwelling older adults, cognitive and exercise training, have been identified as effective non-pharmacological interventions to prevent cognitive decline [22–32]. Exercise training (ET) that generally targets cognitive function [22–24] has been found to have the greatest impact on preserving executive function specifically [25–30]. The impact of ET begins even before improvements in strength and physical functioning are observed [33]. Additionally, cognitive training (CT) has been found to prevent declines in multiple domains of cognitive function, such as executive function, and can impact working memory, abstraction, verbal reasoning, and inhibition [34–42]. It is thought that CT impacts cognitive function by improving neural functions [43–45]. Therefore, multi-domain approaches to CT are preferred over memory training alone. This approach has been associated with broad benefits in cognitive function and provides lasting gains that extend to everyday life activities up to 10 years post-intervention [37], among community-dwelling older adults [40, 46, 47]. CT has also been combined with exercise training for community-dwelling older adults, and this multi-modal approach has been found to be more effective than either intervention alone [43, 48], especially for executive function [33, 39, 48, 49]. We conducted a pilot study of intradialytic ET and CT and found that these interventions preserved cognitive function compared to standard of care (SC) [50].

Therefore, we built upon the findings of our pilot trial of ET and CT to design a trial to test whether these interventions alone or in combination preserve cognitive function among patients initiating hemodialysis. We describe our protocol for the Interventions Made to Preserve Cognitive Function Trial (IMPCT), a randomized controlled trial (RCT) of 200 hemodialysis initiates to test whether intradialytic CT, ET, and combined CT + ET preserves executive function compared to standard of care (SC).

## Methods/design

### Research aims

The primary aim of this trial is to determine if receiving intradialytic CT, ET, or CT + ET preserves executive function relative to those with SC. The secondary objectives are to compare the risk of secondary cognitive outcomes, ESKD-specific clinical outcomes, and patient-centered outcomes among those receiving CT, ET, or CT + ET relative to those in SC. We will accomplish these goals by conducting a multi-dialysis center 2 by 2 factorial RCT (*n* = 200).

### Study setting and team

**Fig. 1:**
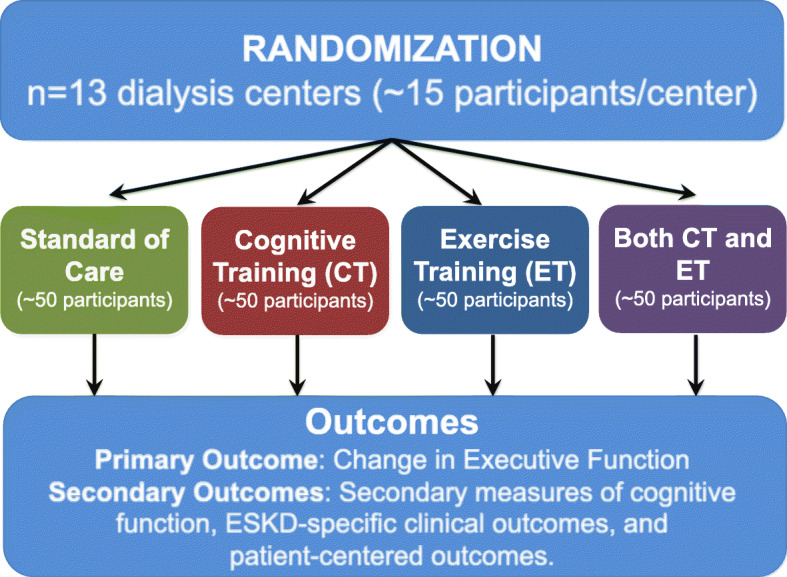
Schema for Interventions Made to Preserve Cognitive Function Trial (IMPCT)

Eligible adult ESKD patients who are initiating twice or thrice weekly maintenance hemodialysis will be recruited from 13 centers in the Baltimore, Maryland area (Fig. 1); additional centers will be added if needed to achieve a sample size of 200 participants. Screening, consent, enrollment, assessments and interventions will be conducted at the dialysis center. A project manager and trained research assistants will conduct all aspects of the clinical trial. Additionally, the study will have a medical monitor who will review all unexpected adverse events and a Data, Safety, and Monitoring Board (DSMB); see below. The DSMB will be comprised of a patient advocate, an ethicist, a statistician, a nephrologist, and a research nurse.

### Participant recruitment

The inclusion and exclusion criteria are listed in Table 1. We will only enroll hemodialysis patients within 3 months to 3 years of hemodialysis initiation because: 1) they will not have already experienced the cognitive decline associated with hemodialysis; 2) there is a high dementia incidence even within the first year of hemodialysis [6]; 3) survival bias is present when studying prevalent hemodialysis patients; and 4) we want to intervene before the hemodialysis-associated neurodegenerative process begins. At each center, all English-speaking hemodialysis patients aged ≥18 years who are within 3 months to 3 years of hemodialysis initiation will be screened for eligibility based on exclusion criteria (Table 1). Only those who are willing to participate in research, based on willingness to sign the consent form and to complete the baseline assessment, will be enrolled and then randomized. Finally, approval by the treating provider at the dialysis center is required prior to enrollment to ensure that the interventions will be safe for the participants.

**Table 1:** Inclusion and exclusion criteria for the Interventions Made to Preserve Cognitive Function Trial (IMPCT)

Inclusion Criteria	Men and women with ESKD receiving maintenance hemodialysis 2–3 times weekly at one of the 13 hemodialysis centers. Participants must also be:
	1. Within 3 months to 3 years of initiating hemodialysis
	2. ≥18 years or older at enrollment
	3. English-speaking
	4. Willing to participate in research
	Approval is required by the treating provider at the dialysis center that the interventions are safe for the patient to be enrolled.
**Exclusion Criteria**	Pregnancy, angina pectoris, chronic lung disease requiring oxygen, musculoskeletal conditions, amputation, orthopedic disorders exacerbated by physical activity, a femoral AV access, legally blind, hepatitis B infection requiring medical isolation, or current incarceration, inability to recognize numbers and letters.

We will exclude participants who are prisoners and those who are unable to participate in ET without assistance. Also, participants with the following conditions that would limit participation will be excluded: pregnancy, angina pectoris, chronic lung disease requiring oxygen, musculoskeletal conditions, lower- or upper-extremity amputation, orthopedic disorders exacerbated by physical activity, femoral AV access, legally blind, inability to recognize numbers and letters, or hepatitis B infection requiring medical isolation (Table 2). Trained research assistants will obtain written informed consent from eligible participants.

**Table 2:** Primary and Secondary Endpoints for the Interventions Made to Preserve Cognitive Function Trial (IMPCT)

Primary Endpoint(s)	3-month change in executive function measured using the Trail Making Test Part A (TMTA) and Part B (TMTB). Executive function is measured as TMTB-TMTA.
**Secondary Endpoint(s)**	1. Secondary cognitive measures (change in)
	a. 6-month executive function (TMTB-TMTA)
	b. 3- and 6-month executive function (additional measures)
	c. 3- and 6-month memory
	d. 3- and 6-month global cognitive function
	2. ESKD-specific clinical measures
	a. Measured physical function
	b. Number of falls
	c. Hospitalization
	d. Mortality
	e. Return to work
	f. Amputation
	3. Patient-centered outcomes
	a. HRQOL
	b. Anxiety
	c. Depression
	d. Fatigue
	e. Pain interference
	f. Perceived physical function
	g. Sleep disturbance
	h. Ability to participate in social roles and activities

We expect to screen 340–350 participants to identify 200 eligible participants to randomize to interventions. We will prematurely terminate a study participant after randomization if they violate study procedures, become too medically unfit to continue, receive kidney transplantation, change dialysis centers to one that is not a part of the study, or request early discontinuation for any other reason.

### Data collection

All data collection is conducted in the dialysis center. Any assessment that may be impacted by the hemodialysis treatment is collected prior to initiation of hemodialysis. Personal information will be collected in private and not be shared to protect confidentiality.

### Baseline assessment

At the baseline assessment (Table 3), participants will self-report demographics including age, sex, race/ethnicity, education, employment status, and marital status. Furthermore, trained research assistants will measure frailty using the physical frailty phenotype [51], functional dependence using activities of daily living [52] as well as instrumental activities of daily living [53], impairment in lower extremity function using the Short Physical Performance Battery [54], comorbidity using the Charlson comorbidity index for ESKD [55, 56], and self-reported HRQOL [57]. Additional pre-hemodialysis medical factors, dialysis factors, and clinical measures will be abstracted from the dialysis medical records. At baseline, measures of primary and secondary outcomes (ascertainment described below), are obtained to be able to calculate change in outcome measures after 3 and 6 months of intervention. All participants completing the baseline assessments will be compensated $10 (funding from NIDDK R01DK114074).

**Table 3:** Baseline and Follow-up Assessments

Assessment	Collection Method	Baseline	3-Month Follow-up	6-Month Follow-up
*Executive function*	Direct measurement	X	X	X
Trail Making Test Parts A and B (TMTA/B)				
Stroop Test				
Digit Symbol Substitution Test				
*Global cognitive function*	Direct measurement	X	X	X
MoCA				
Auditory/Verbal Learning Test	Direct measurement	X	X	X
Physical Frailty Phenotype	Direct measurement	X	X	X
Self-reported Quality of Life	Validated questionnaire	X	X	X
*Lower-extremity function*	Direct measurement	X	X	X
Short Physical Performance Battery				
*Functional Status:*	Self-report	X	X	X
IADL/ADL				
PROMIS-29 short-form profile	Validated self-report instrument	X	X	X
Anxiety, depression, fatigue, pain, perceived function, sleep disturbance, and participation in social roles/activities				
Demographics	Self-report and abstraction	X		
Working status	Self-report	X	X	X
Charlson Comorbidity Index for ESKD	Self-report and abstraction	X	X	X
Health behaviors (Smoking status, alcohol intake, and illicit drug use)	Self-report and abstraction	X	X	X
Medical factors (pre-dialysis factors)	Chart abstraction	X		
Clinical measures	Chart abstraction	X		
Amputation	Self-report and abstraction	X	X	X
Dialysis factors	Chart abstraction	X	X	X

### Randomization

Participants will be randomized after completion of the baseline assessment. Participants will be block randomized by sex, race, and dialysis center. They will be randomly assigned to one of the 4 arms using a blind and secure computer-based allocation system. To assure desired sample sizes in the 4 arms, the randomized block size will also be randomized. We will perform the randomization schema using R. Blinding of intervention groups to participants and clinical staff will not be possible; however, those collecting the primary and secondary outcomes will be blinded to the intervention group.

### Trial interventions

Participants will be asked to participate in CT and ET for a minimum of 30 min during each hemodialysis session. After 15 min on hemodialysis, the research assistant will approach the participant to initiate the intervention. When the participant is finished with their assigned intervention, the study staff will record the duration of the intervention for each session. Additionally, the blood pressure of participants will be recorded before and after each intervention. Participants in the CT, ET, or CT + ET arm will be compensated with a $5 gift card for every intervention session in which they participate so as to encourage participation. We will administer the interventions for 6 months (funding from NIDDK R01DK114074).

### Intradialytic ET

Participants randomized to the ET arm will be given a stationary foot peddler, which will be placed at a distance from the dialysis chair that is comfortable for the participant. The research assistant will adjust the resistance on the foot peddler for each participant.

To standardize the dose of ET, all ET will start with a 2 min warm up, then the resistance will be adjusted so that the participant is working at perceived exertion of “somewhat hard,” using the Borg scale [58] (~ 50 rpm). Resistance will be increased when the rating falls below “somewhat hard.” Heart rate and blood pressure are routinely monitored throughout the session. Participants will participate in ET when their blood pressure is between 110/50–200/100 mmHg and heart rate is < 80% This approach is consistent with previous intradialytic ET [59].

### Intradialytic CT

Intradialytic CT consists of playing brain games through Lumosity®, which is a web-based cognitive training program on a tablet PC for at least 30 min. Lumosity is available for research purposes and has been used for cognitive training interventions across a variety of research settings [60–63]. We chose this intervention because it will be well recognized by participants and was generally regarded as a fun activity during the pilot study. We expect that this will lead to increased participation and adherence. Lumosity has adapted scientifically developed cognitive training tasks into over 40 games. Participants will have 10 different brain games each session to play and these games will vary for each session.

The brain games used in the CT arm are not designed to teach a specific cognitive ability. Therefore, we will test whether there is a transfer of training to global cognition and executive function; see below. It is important to show that we are not just teaching the test, which can occur when the cognitive exercise is the same as the outcome.

### Combined Intradialytic CT + ET

For those in the intradialytic CT + ET arm, participants will start with 30 min of CT with a 15 min break and then 30 min of ET. We chose 30 min of participation in each arm as that was the average duration from the pilot study [50] where participants were asked to partake in these intradialytic interventions for as long as they were able. Research assistants will record the duration of both CT and ET for the participants in this arm.

### Outcome measurements

Outcomes are measured at 3 and 6 months of intervention and will be conducted at the dialysis centers. All staff who perform the baseline and follow-up assessments for the primary and secondary outcomes will be blinded to the treatment group. Participants randomized to CT, ET, or ET + CT will be compensated $10 for the completion of each follow-up assessment; those in the SC arm will be compensated $25 (funding from NIDDK R01DK114074); the compensation is higher because these participants will not receive any compensation for participating in the intervention.

### Primary outcomes

Based on the findings of the pilot study [50], the primary endpoint chose was the 3-month change in executive function; executive function is measured using two tests, the Trail Making Test Part A (TMTA) and Trail Making Test Part B (TMTB) [64]; specifically, executive function is calculated as TMTB-TMTA to account for psychomotor speed. The TMTA and TMTB are validated measures of executive function (i.e. cognitive shifting, cognitive flexibility), attention, concentration, and psychomotor speed [65]. The tests measure the time required to connect a series of sequentially numbered (TMTA) and numbered/lettered (TMTB) circles. Needing more time to complete the tests indicates worse executive function. The times are capped at 3 min for TMTA and 5 min for TMTB.

### Secondary outcomes

The secondary outcomes were: 1) cognitive, 2) ESKD-specific clinical outcomes, and 3) patient-centered outcomes; see below.

The secondary cognitive measures included the 6-month change in executive function. Additional tests of global cognitive function and executive function that are less prone to floor or ceiling effects will be secondary cognitive outcomes. The 3- and 6-month change in executive function will be measured using the Digit Symbol Substitution Test [66] and Stroop test [67]. The Digit Symbol Substitution Test [66] evaluates the speed and working memory components of executive function and consists of 9 number/symbol pairs (for example: 1+, 2X, 3=, etc.). Participants are asked to write the corresponding symbol as quickly as possible (for example: 3_, 1_, 9_). The correct number of symbols within 90 s is measured. The Stroop test (reading, color-naming, and interference sub-tasks) [67] evaluates the inhibitory control of executive function and involves reading the name of a color printed in a different color ink: BLUE. The time ratio of color-word interference and color only tasks will be calculated. The 3- and 6-month changes in global cognitive function will be measured by the Montreal Cognitive Assessment (MoCA) [68], a commonly studied, clinically useful, and validated test of global cognitive function. It is an alternative to the Modified Mini-mental State (3MS) examination and measures cognitive performance across the whole continuum, with higher sensitivity for detecting mild cognitive impairment [68]. Finally, the 3- and 6-month change in memory measured by Auditory/Verbal Learning Test (AVLT) [69], a screening test for memory impairment. The AVLT measures a patient’s immediate recall. A series of unrelated words are presented aloud and participants are asked to recall as many as they can.

Secondary ESKD-specific clinical outcomes include, measured lower extremity function by the Short Physical Performance Battery [54], the number of injurious falls, amputations, and hospitalizations, as well as return to work and mortality.

Secondary patient-centered outcomes include self-reported anxiety, depression, fatigue, pain interference, perceived physical function, sleep disturbance, ability to participate in social roles and activities as well as HRQOL. These patient-reported outcomes will each be measured by 4-items in the Patient-Reported Outcomes Measurement Information System (PROMIS)-29 short form profile from the NIH toolbox [70]. PROMIS is a set of person-centered measures that evaluates physical mental, and social health in adults. The items were developed and validated in a way that is psychometrically sound and relevant across chronic health conditions. Each is measured by 4-items in the PROMIS-29 short form profile. We will measure HRQOL using a self-report of current health and health in the past year.

Participants will be followed for 1 year after the end of the intervention for the secondary outcomes.

### Adverse events

The expected adverse events are cramping (among ET group), hypotension (among ET group), hypertension (among ET group), elevated heart rate (among ET group), or headache (among CT group). During every session, participants in all 4 arms are asked to report whether or not they have experienced any of these expected adverse events.

All unexpected adverse events are ascertained by direct observation, interviewing participants, and unsolicited reports from participants or dialysis staff. The medical monitor and PI will review each unexpected adverse event and classify it by severity (mild, moderate, severe/undesirable, potentially life threatening or death) and by grading (unrelated, possibly related, or definitely related). These adverse events will be recorded from the time of enrollment until a subject completes study participation or until 30 days after he/she withdraws.

### Participant safety

The DSMB will meet to review interim analyses of the safety and efficacy data; meetings will occur after 10, 25, and 50% of participants, respectively, having completed 3 months of the interventions. The DSMB will review any event that potentially impacts safety at the request of the Principal Investigator, or medical monitor. Additionally, the DSMB may be called upon for ad hoc reviews of unexpected adverse events. After the initiation of the trial, the IRB will take action based on recommendations from the DSMB if there are superiority or safety concerns. A temporary halt in enrollment at all participating centers will be implemented if an ad hoc DSMB safety review is required.

### Sample size

We used a Monte-Carlo simulation (1000 randomly generated datasets) to estimate the power of a 2-by-2 factorial design with linear regression and Huber-White robust standard errors. We powered the RCT to detect a statistically significant difference in the CT + ET group based on a 3-month change in executive function, given the group mean change and SD in the pilot study [50].

So that when each arm of the study has 47 participants, we have 80% power to detect a statistically significant change of 20 s between baseline and 3 months comparing the CT + ET arm to the SC arm as well as 95% power to detect a statistically significant change of 15 s in the CT arm and > 99% to detect a statistically significant change of 18 s in the ET arm. Therefore, we would need to recruit 200 participants. Even if we were to recruit just 22 participants (rather than 47) into each arm of the study, we would still have > 80% power to observe statistically significant changes of 15 s for the main effects of CT and ET.

### Statistical analysis

Data will be analyzed according to the intention-to-treat principle. We will test the main effect of CT alone and the main effect of ET alone as well as the interaction between CT + ET compared with SC. All data analyses will be conducted in Stata 14.0 (or higher) software. Statistical significance will be set at *P* < 0.05 for all analyses. Data will be directly entered into RedCap and weekly assessment of data completeness and range checks as well as other standard data management procedures will be used.

Descriptive statistics will include estimates of mean and standard deviations for continuous factors, percentages for categorical factors, and medians and interquartile ranges for non-normally distributed continuous factors. Skewness/kurtosis will be tested to assess the normality of all continuous data; log transformations for skewed distributions will be used. Baseline characteristics will be compared among treatment groups to test adequacy of randomization and identify possible confounders. If there are concerns about imbalances between groups with respect to important risk factors for executive function decline, we will adjust regression-based models as necessary to account for these differences. Competing events (death or transplantation) for the measurement of 3-month change in executive function will be quantified; if relevant, sensitivity analysis accounting for competing events will be performed.

Change in executive function between baseline and 3 months will be handled as a continuous outcome. Using a linear regression with the cluster option to account for the correlation of patients within centers (if needed) and any imbalance between the groups, we will analyze the primary endpoint. We will test for intervention effect measure modification by frailty, age, race, and sex for the primary endpoint as described below. Secondary endpoints will be analyzed based on the functional form of the outcome.

### Trial status

This trial has been approved by the Johns Hopkins IRB and reviewed by Frenova Renal Research and DaVita Clinical Research. All important protocol modifications will be reported to the Johns Hopkins IRB and reviewed by Frenova Renal Research and DaVita Clinical Research. It is registered at clinicaltrials.gov (NCT03616535): https://clinicaltrials.gov/ct2/show/NCT03616535. The trial is currently ongoing with the first patient randomized on September 7, 2018.

## Discussion

Cognitive decline and dementia are two major clinical and public health challenges among patients undergoing hemodialysis. Therefore, we designed a novel 2 by 2 factorial RCT of intradialytic CT and ET among patients initiating hemodialysis (*n* = 200); a defining feature of this study is that all visits and interventions are being conducted in the hemodialysis center.

To date, there have been only two trials that have explored exercise as an intervention with secondary outcomes of cognitive function among patients undergoing dialysis [71, 72]. The first trial was the Exercise Introduction to Enhance Performance in Dialysis (EXCITE) trial, a 6-month randomized, controlled, multicenter trial of adult patients of all ages undergoing dialysis [72]. The goal of this trial was to test whether home-based, personalized exercise interventions improve functional status; this study additionally collected self-reported cognitive function. The EXCITE trial results suggest that exercise improves self-reported cognitive function scores as measured by the Kidney Disease QOL-SF [72]. In a secondary analysis, this trial tested the impact and tolerance of the exercise program on older (aged ≥65 years) dialysis patients. Exercise preserved self-reported cognitive function in this older population; those randomized to the control arm experienced more declines, on average, in self-reported cognitive function [73].

In addition to the EXCITE trial, the second trial was a pilot randomized controlled trial studying intradialytic exercise as an intervention to preserve cognitive function [71]. This first pilot RCT of 30 adult (aged ≥18 years) patients undergoing hemodialysis evaluated the impact of a 4-month exercise intervention of intradialytic cycling and tested whether this aerobic exercise affected cerebral blood flow and cognitive function. The results suggest that those randomized to the exercise group (*N* = 15) had improved cognitive function, a greater proportion of arteries with increased flow velocity, and improved basilar maximum blood flow velocity compared to the control group (N = 15).

Finally, we conducted a pilot randomized controlled trial of 20 adult (aged ≥18 years) patients undergoing hemodialysis and tested whether 3 months of intradialytic CT (tablet PC-based brain games) (*N* = 7), ET (foot peddlers) (*N* = 6), or SC (N = 7) preserved cognitive function [50]. Cognitive function was directly measured using the 3MS,TMTA, and TMTB. While those patients randomized to the SC arm experienced decline in psychomotor speed and executive function at 3 months, those randomized to either the CT or ET arms demonstrated preserved cognitive function.

In sum, the current trial will be an important step in identifying which intradialytic interventions preserve cognitive health among patients undergoing hemodialysis. We will leverage the time spent in a dialysis center to deliver these interventions so that patients will have higher compliance with their assigned intervention and hopefully find that these activities will preserve cognitive function. We will be able to isolate the impact of intradialytic ET and CT on 1) secondary cognitive outcomes, 2) ESKD-specific clinical outcomes, and 3) patient-centered outcomes. Finally, we will test the novel hypothesis that combined intradialytic ET and CT synergistically preserve cognitive function. In light of the growing concern about cognitive decline and dementia among patients undergoing hemodialysis, we hope to provide new information about potential effective interventions to preserve cognitive function for this vulnerable population.

## Data Availability

Data sharing is not applicable to this article as no datasets were generated or analyzed during the current study. Access to the final trial dataset will be available to the PI (Dr. McAdams-DeMarco) by email (mara@jhu.edu). If outside investigators are interested in the randomized controlled trial data collected through the study we will consider applications for collaborative work. The PI can provide information on how to apply for permission to obtain access to the data. Results from the trial will be published in peer reviewed journals and presented at relevant scientific meetings. There are no publication restrictions.

## Abbreviations

AVLT: Auditory/Verbal Learning Test
CT: Cognitive training
DSMB: Data and Safety Monitoring Board
ESKD: End-stage kidney disease
EXCITE: Exercise Introduction to Enhance Performance in Dialysis
ET: Exercise training
HRQOL: Health-related quality of life
IMPCT: Interventions Made to Preserve Cognitive Function Trial
3MS: Modified Mini-mental State
MoCA: Montreal Cognitive Assessment
PROMIS: Patient-Reported Outcomes Measurement Information System
RCT: Randomized controlled trial
TMTA: Trail Making Test Part A
TMTB: Trail Making Test Part B
